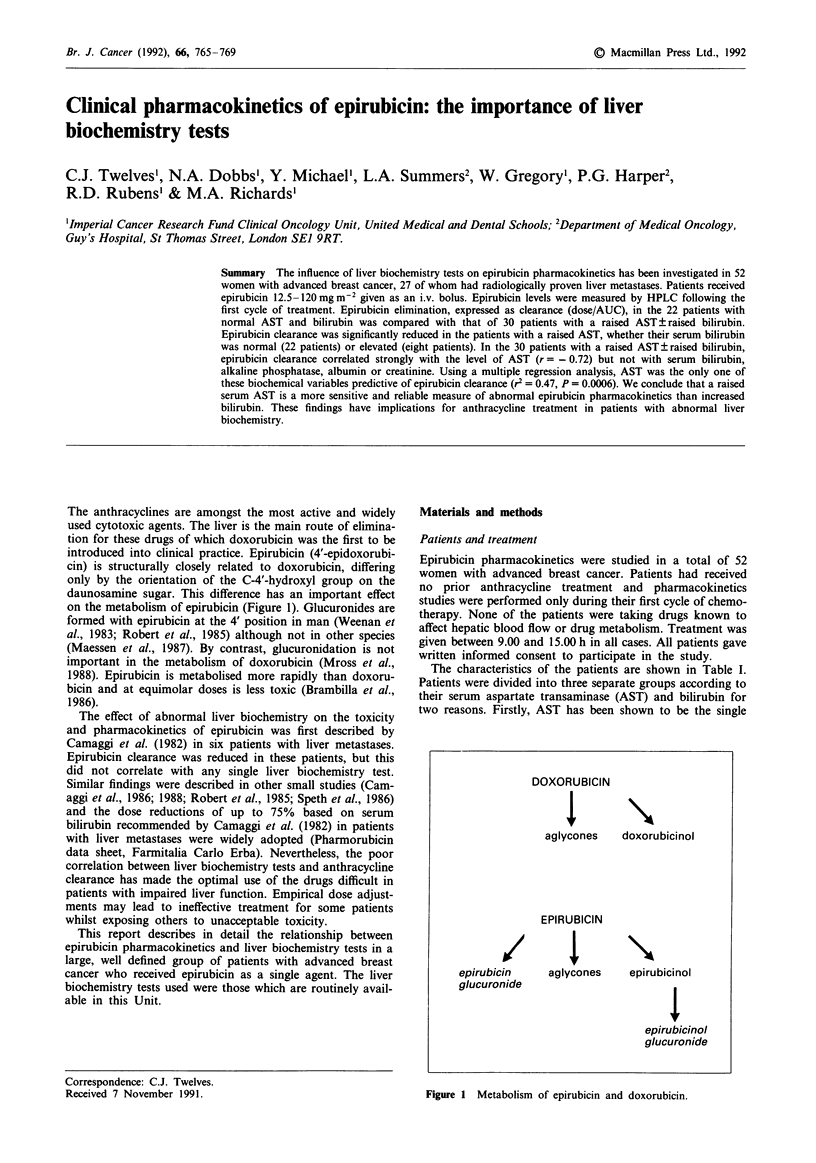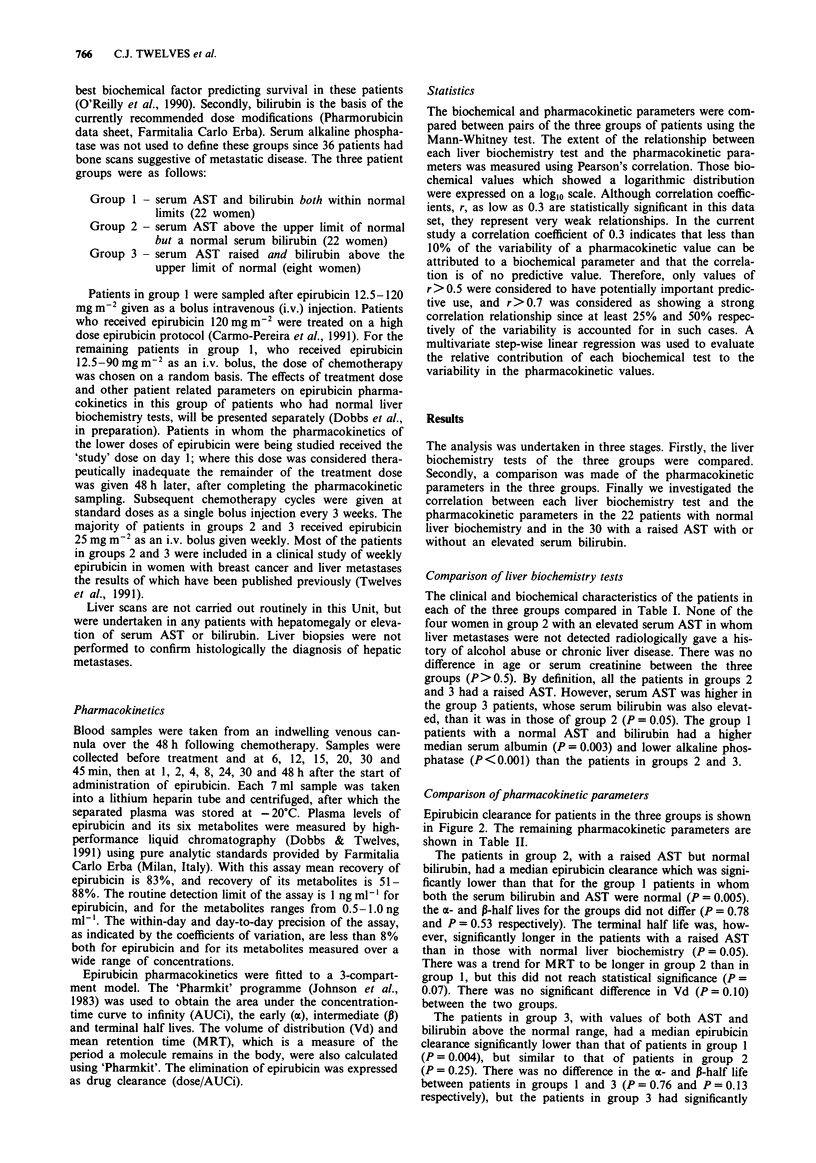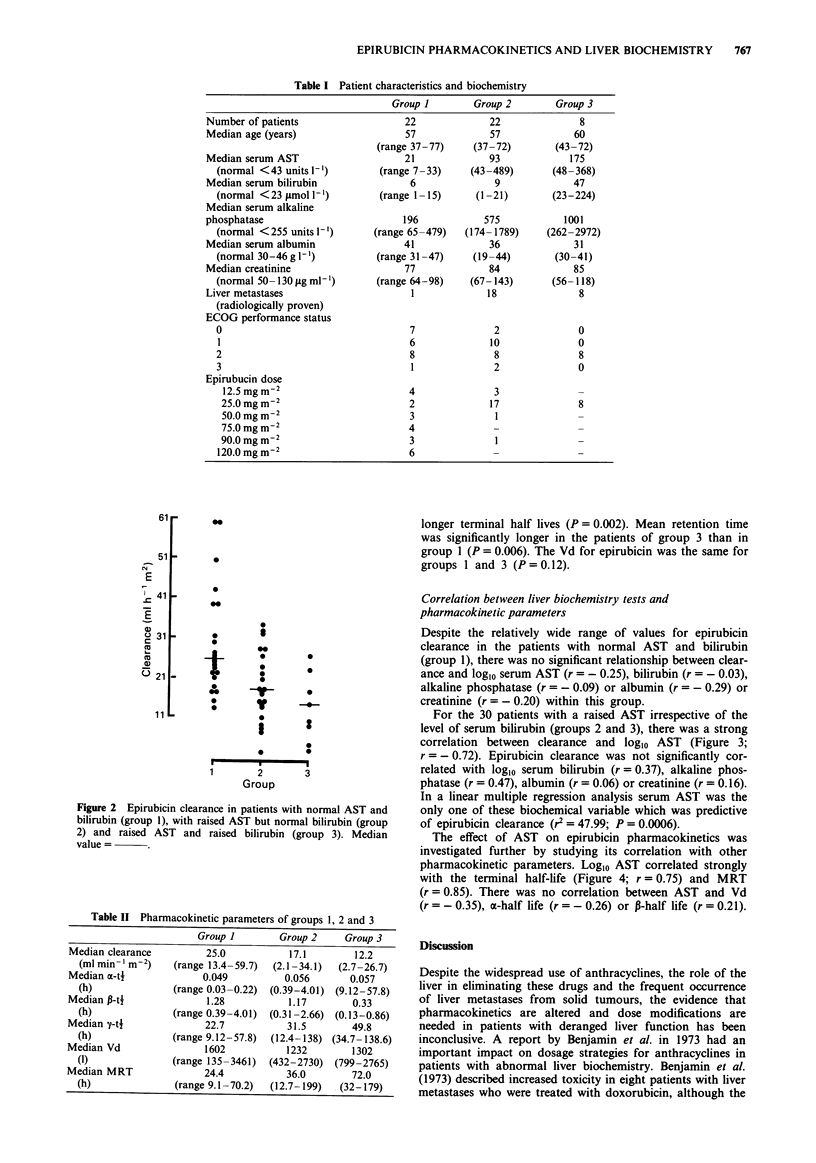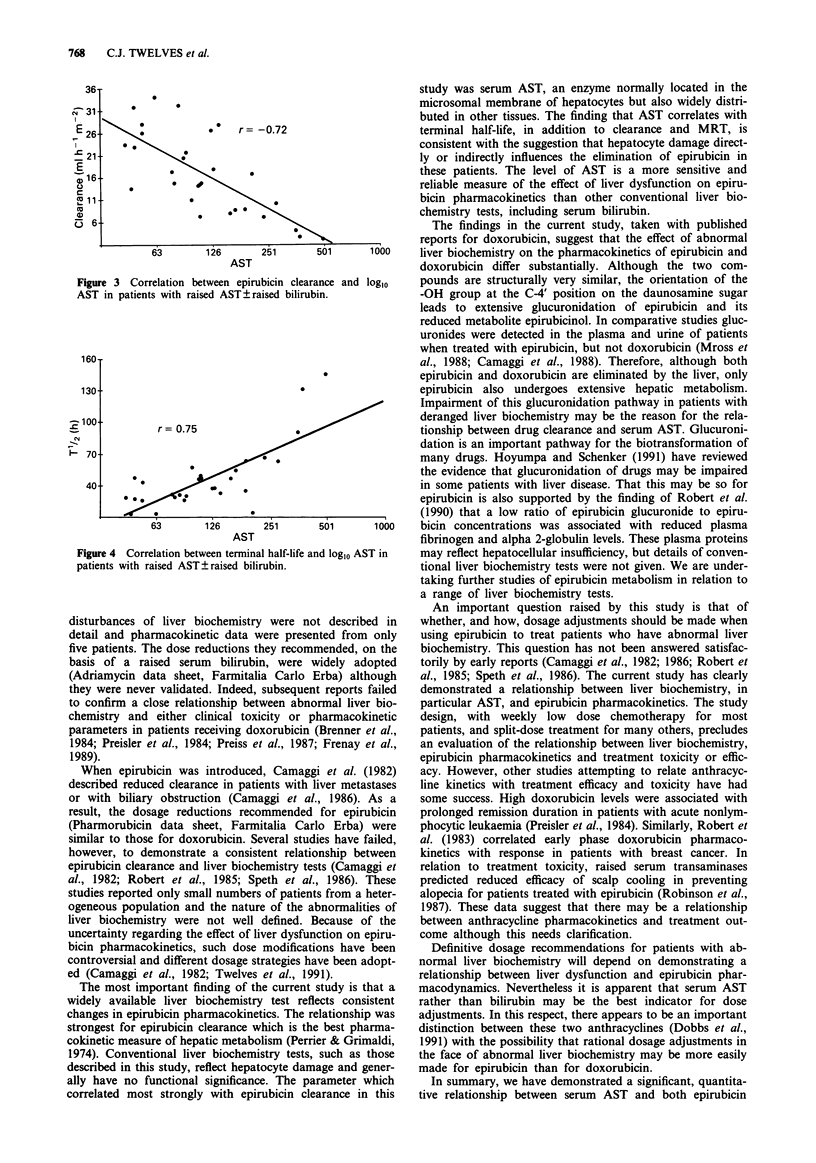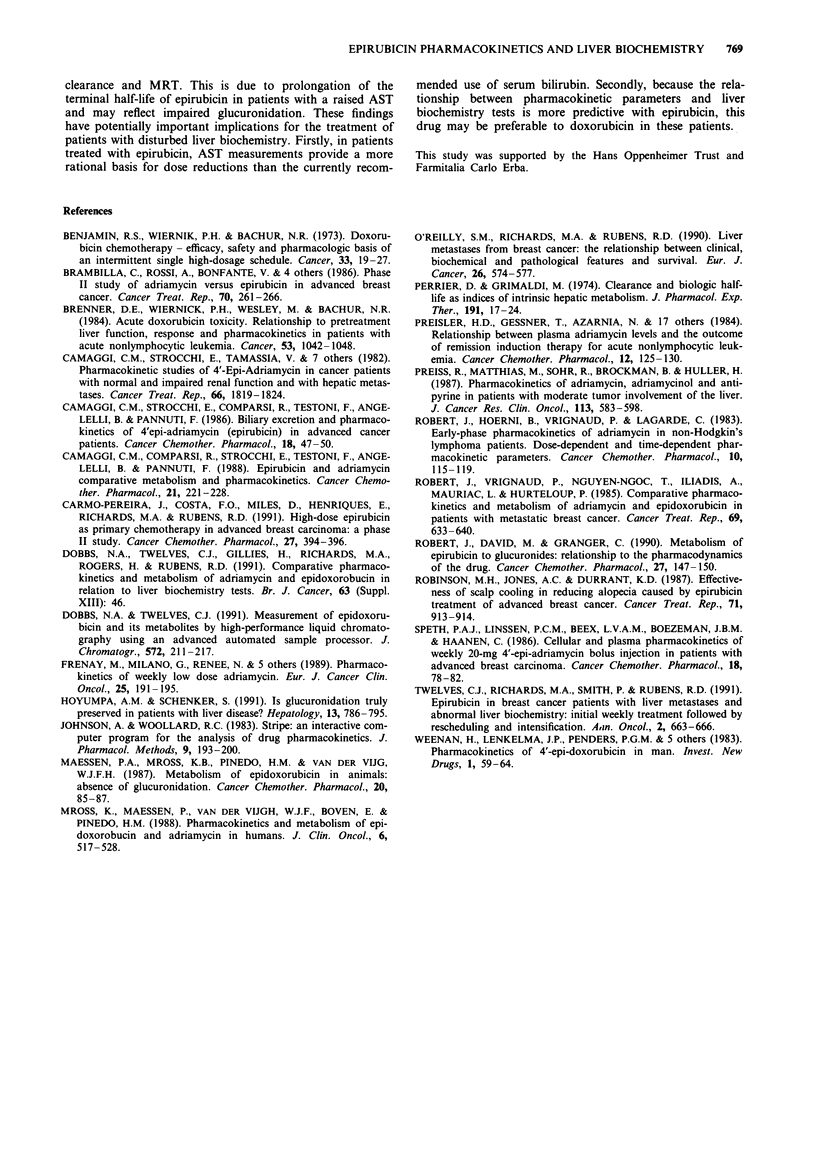# Clinical pharmacokinetics of epirubicin: the importance of liver biochemistry tests.

**DOI:** 10.1038/bjc.1992.353

**Published:** 1992-10

**Authors:** C. J. Twelves, N. A. Dobbs, Y. Michael, L. A. Summers, W. Gregory, P. G. Harper, R. D. Rubens, M. A. Richards

**Affiliations:** Imperial Cancer Research Fund Clinical Oncology Unit, United Medical School, Guy's Hospital, London.

## Abstract

The influence of liver biochemistry tests on epirubicin pharmacokinetics has been investigated in 52 women with advanced breast cancer, 27 of whom had radiologically proven liver metastases. Patients received epirubicin 12.5-120 mg m-2 given as an i.v. bolus. Epirubicin levels were measured by HPLC following the first cycle of treatment. Epirubicin elimination, expressed as clearance (dose/AUC), in the 22 patients with normal AST and bilirubin was compared with that of 30 patients with a raised AST +/- raised bilirubin. Epirubicin clearance was significantly reduced in the patients with a raised AST, whether their serum bilirubin was normal (22 patients) or elevated (eight patients). In the 30 patients with a raised AST +/- raised bilirubin, epirubicin clearance correlated strongly with the level of AST (r = -0.72) but not with serum bilirubin, alkaline phosphatase, albumin or creatinine. Using a multiple regression analysis, AST was the only one of these biochemical variables predictive of epirubicin clearance (r2 = 0.47, P = 0.0006). We conclude that a raised serum AST is a more sensitive and reliable measure of abnormal epirubicin pharmacokinetics than increased bilirubin. These findings have implications for anthracycline treatment in patients with abnormal liver biochemistry.


*Br. J. Cancer* (1992), **66**, 765–769 © Macmillan Press Ltd., 1992

### Clinical pharmacokinetics of epirubicin: the importance of liver biochemistry tests

C.J. Twelves[1], N.A. Dobbs[1], Y. Michael[1], L.A. Summers[2], W. Gregory[1], P.G. Harper[2], R.D. Rubens[1] & M.A. Richards[1]

[1]*Imperial Cancer Research Fund Clinical Oncology Unit, United Medical and Dental Schools;* [2]*Department of Medical Oncology, Guy's Hospital, St Thomas Street, London SE1 9RT.*

**Summary** The influence of liver biochemistry tests on epirubicin pharmacokinetics has been investigated in 52 women with advanced breast cancer, 27 of whom had radiologically proven liver metastases. Patients received epirubicin 12.5–120 mg $m^{-2}$ given as an i.v. bolus. Epirubicin levels were measured by HPLC following the first cycle of treatment. Epirubicin elimination, expressed as clearance (dose/AUC), in the 22 patients with normal AST and bilirubin was compared with that of 30 patients with a raised AST ± raised bilirubin. Epirubicin clearance was significantly reduced in the patients with a raised AST, whether their serum bilirubin was normal (22 patients) or elevated (eight patients). In the 30 patients with a raised AST ± raised bilirubin, epirubicin clearance correlated strongly with the level of AST ($r = -0.72$) but not with serum bilirubin, alkaline phosphatase, albumin or creatinine. Using a multiple regression analysis, AST was the only one of these biochemical variables predictive of epirubicin clearance ($r^2 = 0.47$, $P = 0.0006$). We conclude that a raised serum AST is a more sensitive and reliable measure of abnormal epirubicin pharmacokinetics than increased bilirubin. These findings have implications for anthracycline treatment in patients with abnormal liver biochemistry.

The anthracyclines are amongst the most active and widely used cytotoxic agents. The liver is the main route of elimination for these drugs of which doxorubicin was the first to be introduced into clinical practice. Epirubicin (4′-epidoxorubicin) is structurally closely related to doxorubicin, differing only by the orientation of the C-4′-hydroxyl group on the daunosamine sugar. This difference has an important effect on the metabolism of epirubicin (Figure 1). Glucuronides are formed with epirubicin at the 4′ position in man (Weenan *et al.*, 1983; Robert *et al.*, 1985) although not in other species (Maessen *et al.*, 1987). By contrast, glucuronidation is not important in the metabolism of doxorubicin (Mross *et al.*, 1988). Epirubicin is metabolised more rapidly than doxorubicin and at equimolar doses is less toxic (Brambilla *et al.*, 1986).

The effect of abnormal liver biochemistry on the toxicity and pharmacokinetics of epirubicin was first described by Camaggi *et al.* (1982) in six patients with liver metastases. Epirubicin clearance was reduced in these patients, but this did not correlate with any single liver biochemistry test. Similar findings were described in other small studies (Camaggi *et al.*, 1986; 1988; Robert *et al.*, 1985; Speth *et al.*, 1986) and the dose reductions of up to 75% based on serum bilirubin recommended by Camaggi *et al.* (1982) in patients with liver metastases were widely adopted (Pharmorubicin data sheet, Farmitalia Carlo Erba). Nevertheless, the poor correlation between liver biochemistry tests and anthracycline clearance has made the optimal use of the drugs difficult in patients with impaired liver function. Empirical dose adjustments may lead to ineffective treatment for some patients whilst exposing others to unacceptable toxicity.

This report describes in detail the relationship between epirubicin pharmacokinetics and liver biochemistry tests in a large, well defined group of patients with advanced breast cancer who received epirubicin as a single agent. The liver biochemistry tests used were those which are routinely available in this Unit.

Correspondence: C.J. Twelves.
Received 7 November 1991.

### Materials and methods

### *Patients and treatment*

Epirubicin pharmacokinetics were studied in a total of 52 women with advanced breast cancer. Patients had received no prior anthracycline treatment and pharmacokinetics studies were performed only during their first cycle of chemotherapy. None of the patients were taking drugs known to affect hepatic blood flow or drug metabolism. Treatment was given between 9.00 and 15.00 h in all cases. All patients gave written informed consent to participate in the study.

The characteristics of the patients are shown in Table I. Patients were divided into three separate groups according to their serum aspartate transaminase (AST) and bilirubin for two reasons. Firstly, AST has been shown to be the single

**Figure 1** Metabolism of epirubicin and doxorubicin.

best biochemical factor predicting survival in these patients (O'Reilly et al., 1990). Secondly, bilirubin is the basis of the currently recommended dose modifications (Pharmorubicin data sheet, Farmitalia Carlo Erba). Serum alkaline phosphatase was not used to define these groups since 36 patients had bone scans suggestive of metastatic disease. The three patient groups were as follows:

Group 1 – serum AST and bilirubin *both* within normal limits (22 women)
Group 2 – serum AST above the upper limit of normal *but* a normal serum bilirubin (22 women)
Group 3 – serum AST raised *and* bilirubin above the upper limit of normal (eight women)

Patients in group 1 were sampled after epirubicin 12.5–120 mg $m^{-2}$ given as a bolus intravenous (i.v.) injection. Patients who received epirubicin 120 mg $m^{-2}$ were treated on a high dose epirubicin protocol (Carmo-Pereira et al., 1991). For the remaining patients in group 1, who received epirubicin 12.5–90 mg $m^{-2}$ as an i.v. bolus, the dose of chemotherapy was chosen on a random basis. The effects of treatment dose and other patient related parameters on epirubicin pharmacokinetics in this group of patients who had normal liver biochemistry tests, will be presented separately (Dobbs et al., in preparation). Patients in whom the pharmacokinetics of the lower doses of epirubicin were being studied received the 'study' dose on day 1; where this dose was considered therapeutically inadequate the remainder of the treatment dose was given 48 h later, after completing the pharmacokinetic sampling. Subsequent chemotherapy cycles were given at standard doses as a single bolus injection every 3 weeks. The majority of patients in groups 2 and 3 received epirubicin 25 mg $m^{-2}$ as an i.v. bolus given weekly. Most of the patients in groups 2 and 3 were included in a clinical study of weekly epirubicin in women with breast cancer and liver metastases the results of which have been published previously (Twelves et al., 1991).

Liver scans are not carried out routinely in this Unit, but were undertaken in any patients with hepatomegaly or elevation of serum AST or bilirubin. Liver biopsies were not performed to confirm histologically the diagnosis of hepatic metastases.

### *Pharmacokinetics*

Blood samples were taken from an indwelling venous cannula over the 48 h following chemotherapy. Samples were collected before treatment and at 6, 12, 15, 20, 30 and 45 min, then at 1, 2, 4, 8, 24, 30 and 48 h after the start of administration of epirubicin. Each 7 ml sample was taken into a lithium heparin tube and centrifuged, after which the separated plasma was stored at −20°C. Plasma levels of epirubicin and its six metabolites were measured by high-performance liquid chromatography (Dobbs & Twelves, 1991) using pure analytic standards provided by Farmitalia Carlo Erba (Milan, Italy). With this assay mean recovery of epirubicin is 83%, and recovery of its metabolites is 51–88%. The routine detection limit of the assay is 1 ng $ml^{-1}$ for epirubicin, and for the metabolites ranges from 0.5–1.0 ng $ml^{-1}$. The within-day and day-to-day precision of the assay, as indicated by the coefficients of variation, are less than 8% both for epirubicin and for its metabolites measured over a wide range of concentrations.

Epirubicin pharmacokinetics were fitted to a 3-compartment model. The 'Pharmkit' programme (Johnson et al., 1983) was used to obtain the area under the concentration-time curve to infinity (AUCi), the early (α), intermediate (β) and terminal half lives. The volume of distribution (Vd) and mean retention time (MRT), which is a measure of the period a molecule remains in the body, were also calculated using 'Pharmkit'. The elimination of epirubicin was expressed as drug clearance (dose/AUCi).

### *Statistics*

The biochemical and pharmacokinetic parameters were compared between pairs of the three groups of patients using the Mann-Whitney test. The extent of the relationship between each liver biochemistry test and the pharmacokinetic parameters was measured using Pearson's correlation. Those biochemical values which showed a logarithmic distribution were expressed on a $\log_{10}$ scale. Although correlation coefficients, $r$, as low as 0.3 are statistically significant in this data set, they represent very weak relationships. In the current study a correlation coefficient of 0.3 indicates that less than 10% of the variability of a pharmacokinetic value can be attributed to a biochemical parameter and that the correlation is of no predictive value. Therefore, only values of $r > 0.5$ were considered to have potentially important predictive use, and $r > 0.7$ was considered as showing a strong correlation relationship since at least 25% and 50% respectively of the variability is accounted for in such cases. A multivariate step-wise linear regression was used to evaluate the relative contribution of each biochemical test to the variability in the pharmacokinetic values.

### Results

The analysis was undertaken in three stages. Firstly, the liver biochemistry tests of the three groups were compared. Secondly, a comparison was made of the pharmacokinetic parameters in the three groups. Finally we investigated the correlation between each liver biochemistry test and the pharmacokinetic parameters in the 22 patients with normal liver biochemistry and in the 30 with a raised AST with or without an elevated serum bilirubin.

### *Comparison of liver biochemistry tests*

The clinical and biochemical characteristics of the patients in each of the three groups compared in Table I. None of the four women in group 2 with an elevated serum AST in whom liver metastases were not detected radiologically gave a history of alcohol abuse or chronic liver disease. There was no difference in age or serum creatinine between the three groups ($P > 0.5$). By definition, all the patients in groups 2 and 3 had a raised AST. However, serum AST was higher in the group 3 patients, whose serum bilirubin was also elevated, than it was in those of group 2 ($P = 0.05$). The group 1 patients with a normal AST and bilirubin had a higher median serum albumin ($P = 0.003$) and lower alkaline phosphatase ($P < 0.001$) than the patients in groups 2 and 3.

### *Comparison of pharmacokinetic parameters*

Epirubicin clearance for patients in the three groups is shown in Figure 2. The remaining pharmacokinetic parameters are shown in Table II.

The patients in group 2, with a raised AST but normal bilirubin, had a median epirubicin clearance which was significantly lower than that for the group 1 patients in whom both the serum bilirubin and AST were normal ($P = 0.005$). the α- and β-half lives for the groups did not differ ($P = 0.78$ and $P = 0.53$ respectively). The terminal half life was, however, significantly longer in the patients with a raised AST than in those with normal liver biochemistry ($P = 0.05$). There was a trend for MRT to be longer in group 2 than in group 1, but this did not reach statistical significance ($P = 0.07$). There was no significant difference in Vd ($P = 0.10$) between the two groups.

The patients in group 3, with values of both AST and bilirubin above the normal range, had a median epirubicin clearance significantly lower than that of patients in group 1 ($P = 0.004$), but similar to that of patients in group 2 ($P = 0.25$). There was no difference in the α- and β-half life between patients in groups 1 and 3 ($P = 0.76$ and $P = 0.13$ respectively), but the patients in group 3 had significantly

**Table I** Patient characteristics and biochemistry

| | *Group 1* | *Group 2* | *Group 3* |
|---|---|---|---|
| Number of patients | 22 | 22 | 8 |
| Median age (years) | 57 | 57 | 60 |
| | (range 37–77) | (37–72) | (43–72) |
| Median serum AST | 21 | 93 | 175 |
| (normal <43 units $l^{-1}$) | (range 7–33) | (43–489) | (48–368) |
| Median serum bilirubin | 6 | 9 | 47 |
| (normal <23 μmol $l^{-1}$) | (range 1–15) | (1–21) | (23–224) |
| Median serum alkaline phosphatase | 196 | 575 | 1001 |
| (normal <255 units $l^{-1}$) | (range 65–479) | (174–1789) | (262–2972) |
| Median serum albumin | 41 | 36 | 31 |
| (normal 30–46 g $l^{-1}$) | (range 31–47) | (19–44) | (30–41) |
| Median creatinine | 77 | 84 | 85 |
| (normal 50–130 μg $ml^{-1}$) | (range 64–98) | (67–143) | (56–118) |
| Liver metastases (radiologically proven) | 1 | 18 | 8 |
| ECOG performance status | | | |
| 0 | 7 | 2 | 0 |
| 1 | 6 | 10 | 0 |
| 2 | 8 | 8 | 8 |
| 3 | 1 | 2 | 0 |
| Epirubucin dose | | | |
| 12.5 mg $m^{-2}$ | 4 | 3 | – |
| 25.0 mg $m^{-2}$ | 2 | 17 | 8 |
| 50.0 mg $m^{-2}$ | 3 | 1 | – |
| 75.0 mg $m^{-2}$ | 4 | – | – |
| 90.0 mg $m^{-2}$ | 3 | 1 | – |
| 120.0 mg $m^{-2}$ | 6 | – | – |

**Figure 2** Epirubicin clearance in patients with normal AST and bilirubin (group 1), with raised AST but normal bilirubin (group 2) and raised AST and raised bilirubin (group 3). Median value = ———.

**Table II** Pharmacokinetic parameters of groups 1, 2 and 3

| | *Group 1* | *Group 2* | *Group 3* |
|---|---|---|---|
| Median clearance | 25.0 | 17.1 | 12.2 |
| (ml $min^{-1}$ $m^{-2}$) | (range 13.4–59.7) | (2.1–34.1) | (2.7–26.7) |
| Median α-t½ | 0.049 | 0.056 | 0.057 |
| (h) | (range 0.03–0.22) | (0.39–4.01) | (9.12–57.8) |
| Median β-t½ | 1.28 | 1.17 | 0.33 |
| (h) | (range 0.39–4.01) | (0.31–2.66) | (0.13–0.86) |
| Median γ-t½ | 22.7 | 31.5 | 49.8 |
| (h) | (range 9.12–57.8) | (12.4–138) | (34.7–138.6) |
| Median Vd | 1602 | 1232 | 1302 |
| (l) | (range 135–3461) | (432–2730) | (799–2765) |
| Median MRT | 24.4 | 36.0 | 72.0 |
| (h) | (range 9.1–70.2) | (12.7–199) | (32–179) |

longer terminal half lives ($P = 0.002$). Mean retention time was significantly longer in the patients of group 3 than in group 1 ($P = 0.006$). The Vd for epirubicin was the same for groups 1 and 3 ($P = 0.12$).

*Correlation between liver biochemistry tests and pharmacokinetic parameters*

Despite the relatively wide range of values for epirubicin clearance in the patients with normal AST and bilirubin (group 1), there was no significant relationship between clearance and $\log_{10}$ serum AST ($r = -0.25$), bilirubin ($r = -0.03$), alkaline phosphatase ($r = -0.09$) or albumin ($r = -0.29$) or creatinine ($r = -0.20$) within this group.

For the 30 patients with a raised AST irrespective of the level of serum bilirubin (groups 2 and 3), there was a strong correlation between clearance and $\log_{10}$ AST (Figure 3; $r = -0.72$). Epirubicin clearance was not significantly correlated with $\log_{10}$ serum bilirubin ($r = 0.37$), alkaline phosphatase ($r = 0.47$), albumin ($r = 0.06$) or creatinine ($r = 0.16$). In a linear multiple regression analysis serum AST was the only one of these biochemical variable which was predictive of epirubicin clearance ($r^2 = 47.99$; $P = 0.0006$).

The effect of AST on epirubicin pharmacokinetics was investigated further by studying its correlation with other pharmacokinetic parameters. $\text{Log}_{10}$ AST correlated strongly with the terminal half-life (Figure 4; $r = 0.75$) and MRT ($r = 0.85$). There was no correlation between AST and Vd ($r = -0.35$), α-half life ($r = -0.26$) or β-half life ($r = 0.21$).

### Discussion

Despite the widespread use of anthracyclines, the role of the liver in eliminating these drugs and the frequent occurrence of liver metastases from solid tumours, the evidence that pharmacokinetics are altered and dose modifications are needed in patients with deranged liver function has been inconclusive. A report by Benjamin *et al.* in 1973 had an important impact on dosage strategies for anthracyclines in patients with abnormal liver biochemistry. Benjamin *et al.* (1973) described increased toxicity in eight patients with liver metastases who were treated with doxorubicin, although the

**Figure 3** Correlation between epirubicin clearance and $\log_{10}$ AST in patients with raised AST ± raised bilirubin.

**Figure 4** Correlation between terminal half-life and $\log_{10}$ AST in patients with raised AST ± raised bilirubin.

disturbances of liver biochemistry were not described in detail and pharmacokinetic data were presented from only five patients. The dose reductions they recommended, on the basis of a raised serum bilirubin, were widely adopted (Adriamycin data sheet, Farmitalia Carlo Erba) although they were never validated. Indeed, subsequent reports failed to confirm a close relationship between abnormal liver biochemistry and either clinical toxicity or pharmacokinetic parameters in patients receiving doxorubicin (Brenner *et al*., 1984; Preisler *et al*., 1984; Preiss *et al*., 1987; Frenay *et al*., 1989).

When epirubicin was introduced, Camaggi *et al*. (1982) described reduced clearance in patients with liver metastases or with biliary obstruction (Camaggi *et al*., 1986). As a result, the dosage reductions recommended for epirubicin (Pharmorubicin data sheet, Farmitalia Carlo Erba) were similar to those for doxorubicin. Several studies have failed, however, to demonstrate a consistent relationship between epirubicin clearance and liver biochemistry tests (Camaggi *et al*., 1982; Robert *et al*., 1985; Speth *et al*., 1986). These studies reported only small numbers of patients from a heterogeneous population and the nature of the abnormalities of liver biochemistry were not well defined. Because of the uncertainty regarding the effect of liver dysfunction on epirubicin pharmacokinetics, such dose modifications have been controversial and different dosage strategies have been adopted (Camaggi *et al*., 1982; Twelves *et al*., 1991).

The most important finding of the current study is that a widely available liver biochemistry test reflects consistent changes in epirubicin pharmacokinetics. The relationship was strongest for epirubicin clearance which is the best pharmacokinetic measure of hepatic metabolism (Perrier & Grimaldi, 1974). Conventional liver biochemistry tests, such as those described in this study, reflect hepatocyte damage and generally have no functional significance. The parameter which correlated most strongly with epirubicin clearance in this study was serum AST, an enzyme normally located in the microsomal membrane of hepatocytes but also widely distributed in other tissues. The finding that AST correlates with terminal half-life, in addition to clearance and MRT, is consistent with the suggestion that hepatocyte damage directly or indirectly influences the elimination of epirubicin in these patients. The level of AST is a more sensitive and reliable measure of the effect of liver dysfunction on epirubicin pharmacokinetics than other conventional liver biochemistry tests, including serum bilirubin.

The findings in the current study, taken with published reports for doxorubicin, suggest that the effect of abnormal liver biochemistry on the pharmacokinetics of epirubicin and doxorubicin differ substantially. Although the two compounds are structurally very similar, the orientation of the -OH group at the C-4′ position on the daunosamine sugar leads to extensive glucuronidation of epirubicin and its reduced metabolite epirubicinol. In comparative studies glucuronides were detected in the plasma and urine of patients when treated with epirubicin, but not doxorubicin (Mross *et al*., 1988; Camaggi *et al*., 1988). Therefore, although both epirubicin and doxorubicin are eliminated by the liver, only epirubicin also undergoes extensive hepatic metabolism. Impairment of this glucuronidation pathway in patients with deranged liver biochemistry may be the reason for the relationship between drug clearance and serum AST. Glucuronidation is an important pathway for the biotransformation of many drugs. Hoyumpa and Schenker (1991) have reviewed the evidence that glucuronidation of drugs may be impaired in some patients with liver disease. That this may be so for epirubicin is also supported by the finding of Robert *et al*. (1990) that a low ratio of epirubicin glucuronide to epirubicin concentrations was associated with reduced plasma fibrinogen and alpha 2-globulin levels. These plasma proteins may reflect hepatocellular insufficiency, but details of conventional liver biochemistry tests were not given. We are undertaking further studies of epirubicin metabolism in relation to a range of liver biochemistry tests.

An important question raised by this study is that of whether, and how, dosage adjustments should be made when using epirubicin to treat patients who have abnormal liver biochemistry. This question has not been answered satisfactorily by early reports (Camaggi *et al*., 1982; 1986; Robert *et al*., 1985; Speth *et al*., 1986). The current study has clearly demonstrated a relationship between liver biochemistry, in particular AST, and epirubicin pharmacokinetics. The study design, with weekly low dose chemotherapy for most patients, and split-dose treatment for many others, precludes an evaluation of the relationship between liver biochemistry, epirubicin pharmacokinetics and treatment toxicity or efficacy. However, other studies attempting to relate anthracycline kinetics with treatment efficacy and toxicity have had some success. High doxorubicin levels were associated with prolonged remission duration in patients with acute nonlymphocytic leukaemia (Preisler *et al*., 1984). Similarly, Robert *et al*. (1983) correlated early phase doxorubicin pharmacokinetics with response in patients with breast cancer. In relation to treatment toxicity, raised serum transaminases predicted reduced efficacy of scalp cooling in preventing alopecia for patients treated with epirubicin (Robinson *et al*., 1987). These data suggest that there may be a relationship between anthracycline pharmacokinetics and treatment outcome although this needs clarification.

Definitive dosage recommendations for patients with abnormal liver biochemistry will depend on demonstrating a relationship between liver dysfunction and epirubicin pharmacodynamics. Nevertheless it is apparent that serum AST rather than bilirubin may be the best indicator for dose adjustments. In this respect, there appears to be an important distinction between these two anthracyclines (Dobbs *et al*., 1991) with the possibility that rational dosage adjustments in the face of abnormal liver biochemistry may be more easily made for epirubicin than for doxorubicin.

In summary, we have demonstrated a significant, quantitative relationship between serum AST and both epirubicin clearance and MRT. This is due to prolongation of the terminal half-life of epirubicin in patients with a raised AST and may reflect impaired glucuronidation. These findings have potentially important implications for the treatment of patients with disturbed liver biochemistry. Firstly, in patients treated with epirubicin, AST measurements provide a more rational basis for dose reductions than the currently recommended use of serum bilirubin. Secondly, because the relationship between pharmacokinetic parameters and liver biochemistry tests is more predictive with epirubicin, this drug may be preferable to doxorubicin in these patients.

This study was supported by the Hans Oppenheimer Trust and Farmitalia Carlo Erba.

### References

BENJAMIN, R.S., WIERNIK, P.H. & BACHUR, N.R. (1973). Doxorubicin chemotherapy – efficacy, safety and pharmacologic basis of an intermittent single high-dosage schedule. *Cancer*, **33**, 19–27.

BRAMBILLA, C., ROSSI, A., BONFANTE, V. & 4 others (1986). Phase II study of adriamycin versus epirubicin in advanced breast cancer. *Cancer Treat. Rep.*, **70**, 261–266.

BRENNER, D.E., WIERNICK, P.H., WESLEY, M. & BACHUR, N.R. (1984). Acute doxorubicin toxicity. Relationship to pretreatment liver function, response and pharmacokinetics in patients with acute nonlymphocytic leukemia. *Cancer*, **53**, 1042–1048.

CAMAGGI, C.M., STROCCHI, E., TAMASSIA, V. & 7 others (1982). Pharmacokinetic studies of 4'-Epi-Adriamycin in cancer patients with normal and impaired renal function and with hepatic metastases. *Cancer Treat. Rep.*, **66**, 1819–1824.

CAMAGGI, C.M., STROCCHI, E., COMPARSI, R., TESTONI, F., ANGELELLI, B. & PANNUTI, F. (1986). Biliary excretion and pharmacokinetics of 4'epi-adriamycin (epirubicin) in advanced cancer patients. *Cancer Chemother. Pharmacol.*, **18**, 47–50.

CAMAGGI, C.M., COMPARSI, R., STROCCHI, E., TESTONI, F., ANGELELLI, B. & PANNUTI, F. (1988). Epirubicin and adriamycin comparative metabolism and pharmacokinetics. *Cancer Chemother. Pharmacol.*, **21**, 221–228.

CARMO-PEREIRA, J., COSTA, F.O., MILES, D., HENRIQUES, E., RICHARDS, M.A. & RUBENS, R.D. (1991). High-dose epirubicin as primary chemotherapy in advanced breast carcinoma: a phase II study. *Cancer Chemother. Pharmacol.*, **27**, 394–396.

DOBBS, N.A., TWELVES, C.J., GILLIES, H., RICHARDS, M.A., ROGERS, H. & RUBENS, R.D. (1991). Comparative pharmacokinetics and metabolism of adriamycin and epidoxorobucin in relation to liver biochemistry tests. *Br. J. Cancer*, **63** (Suppl. XIII): 46.

DOBBS, N.A. & TWELVES, C.J. (1991). Measurement of epidoxorubicin and its metabolites by high-performance liquid chromatography using an advanced automated sample processor. *J. Chromatogr.*, **572**, 211–217.

FRENAY, M., MILANO, G., RENEE, N. & 5 others (1989). Pharmacokinetics of weekly low dose adriamycin. *Eur. J. Cancer Clin. Oncol.*, **25**, 191–195.

HOYUMPA, A.M. & SCHENKER, S. (1991). Is glucuronidation truly preserved in patients with liver disease? *Hepatology*, **13**, 786–795.

JOHNSON, A. & WOOLLARD, R.C. (1983). Stripe: an interactive computer program for the analysis of drug pharmacokinetics. *J. Pharmacol. Methods*, **9**, 193–200.

MAESSEN, P.A., MROSS, K.B., PINEDO, H.M. & VAN DER VIJG, W.J.F.H. (1987). Metabolism of epidoxorubicin in animals: absence of glucuronidation. *Cancer Chemother. Pharmacol.*, **20**, 85–87.

MROSS, K., MAESSEN, P., VAN DER VIJGH, W.J.F., BOVEN, E. & PINEDO, H.M. (1988). Pharmacokinetics and metabolism of epidoxorobucin and adriamycin in humans. *J. Clin. Oncol.*, **6**, 517–528.

O'REILLY, S.M., RICHARDS, M.A. & RUBENS, R.D. (1990). Liver metastases from breast cancer: the relationship between clinical, biochemical and pathological features and survival. *Eur. J. Cancer*, **26**, 574–577.

PERRIER, D. & GRIMALDI, M. (1974). Clearance and biologic half-life as indices of intrinsic hepatic metabolism. *J. Pharmacol. Exp. Ther.*, **191**, 17–24.

PREISLER, H.D., GESSNER, T., AZARNIA, N. & 17 others (1984). Relationship between plasma adriamycin levels and the outcome of remission induction therapy for acute nonlymphocytic leukemia. *Cancer Chemother. Pharmacol.*, **12**, 125–130.

PREISS, R., MATTHIAS, M., SOHR, R., BROCKMAN, B. & HULLER, H. (1987). Pharmacokinetics of adriamycin, adriamycinol and antipyrine in patients with moderate tumor involvement of the liver. *J. Cancer Res. Clin. Oncol.*, **113**, 583–598.

ROBERT, J., HOERNI, B., VRIGNAUD, P. & LAGARDE, C. (1983). Early-phase pharmacokinetics of adriamycin in non-Hodgkin's lymphoma patients. Dose-dependent and time-dependent pharmacokinetic parameters. *Cancer Chemother. Pharmacol.*, **10**, 115–119.

ROBERT, J., VRIGNAUD, P., NGUYEN-NGOC, T., ILIADIS, A., MAURIAC, L. & HURTELOUP, P. (1985). Comparative pharmacokinetics and metabolism of adriamycin and epidoxorubicin in patients with metastatic breast cancer. *Cancer Treat. Rep.*, **69**, 633–640.

ROBERT, J., DAVID, M. & GRANGER, C. (1990). Metabolism of epirubicin to glucuronides: relationship to the pharmacodynamics of the drug. *Cancer Chemother. Pharmacol.*, **27**, 147–150.

ROBINSON, M.H., JONES, A.C. & DURRANT, K.D. (1987). Effectiveness of scalp cooling in reducing alopecia caused by epirubicin treatment of advanced breast cancer. *Cancer Treat. Rep.*, **71**, 913–914.

SPETH, P.A.J., LINSSEN, P.C.M., BEEX, L.V.A.M., BOEZEMAN, J.B.M. & HAANEN, C. (1986). Cellular and plasma pharmacokinetics of weekly 20-mg 4'-epi-adriamycin bolus injection in patients with advanced breast carcinoma. *Cancer Chemother. Pharmacol.*, **18**, 78–82.

TWELVES, C.J., RICHARDS, M.A., SMITH, P. & RUBENS, R.D. (1991). Epirubicin in breast cancer patients with liver metastases and abnormal liver biochemistry: initial weekly treatment followed by rescheduling and intensification. *Ann. Oncol.*, **2**, 663–666.

WEENAN, H., LENKELMA, J.P., PENDERS, P.G.M. & 5 others (1983). Pharmacokinetics of 4'-epi-doxorubicin in man. *Invest. New Drugs*, **1**, 59–64.